# Revision of the phylogeny and chorology of the tribe Iphisini with the revalidation of *Colobosaura kraepelini* Werner, 1910 (Reptilia, Squamata, Gymnophthalmidae)

**DOI:** 10.3897/zookeys.669.12245

**Published:** 2017-04-21

**Authors:** Pier Cacciali, Nicolás Martínez, Gunther Köhler

**Affiliations:** 1 Senckenberg Forschungsinstitut und Naturmuseum, Senckenberganlage 25, 60325 Frankfurt a.M., Germany; 2 Goethe-University, Institute for Ecology, Evolution & Diversity, Biologicum, Building C, Max-von-Laue-Str. 13, 60438 Frankfurt am Main, Germany; 3 Instituto de Investigación Biológica del Paraguay, Del Escudo 1607, Asunción, Paraguay; 4 Museo Nacional de Historia Natural del Paraguay. 2169 CDP, Sucursal 1, Ciudad Universitaria, San Lorenzo, Paraguay

**Keywords:** 16S barcodes, Humid Chaco, neotype, Paraguay, taxonomy

## Abstract

The family Gymnophthalmidae contains nearly 235 species with a distribution range from southern Mexico to central Argentina as well as in the Antilles. Among gymnophthalmids, the genus *Colobosaura* is a member of the tribe Iphisini, and currently is considered monotypic (*C. modesta*). The diversity of the tribe was studied recently, with the erection of several new genera. In this work genetic and morphological data of specimens of *Colobosaura* recently collected in Paraguay were analyzed. Genetic (16S barcode) data indicate that these samples are not conspecific with *C. modesta* and they are allocated to the nominal species *C. kraepelini*. Because the original primary type of the latter taxon is considered to be lost, a neotype (SMF 101370) is designated for this species and a redescription provided based on our material. *Colobosaura kraepelini* is distributed in the Humid Chaco, being the only member of the whole tribe in this ecoregion.

## Introduction

Gymnophthalmids are among the least known Neotropical lizards given their secretive habits and small size, and some of them are known only from the original description (Castoe et al. 2004). Currently, 232 species of gymnophthalmid lizards are recognized (Goicochea et al. 2016) with a geographic distribution ranging from Argentina widely across South America to southern Mexico, including some Caribbean islands (Doan 2003, Vitt and Caldwell 2009), with several recently described taxa from the Caatinga and the Cerrado (Ribeiro Delfim et al. 2006). In fact, Cacciali (2010) pointed out the high diversity of gymnophthalmid lizards in the Paraguayan Cerrado with respect to other ecoregions in the country.

In the last decade, this family has been analyzed from a molecular perspective, leading to some changes in phylogenetic hypotheses (Castoe et al. 2004, Rodrigues et al. 2007, Peloso et al. 2011).

One of the genera that underwent taxonomic modifications is *Colobosaura*, which was established by Boulenger (1887) to include *Perodactylus modestus* Reinhardt & Lütken, 1862 described from Morro da Garça, Minas Gerais, Brazil. Somewhat later, Werner (1910) described *Perodactylus kraepelini* from Puerto Max, Concepción, Paraguay. Amaral (1933) considered *C. kraepelini* to be a synonym of *C. modesta* attributing the observed morphological variation to sexual dimorphism. In that contribution the author described *Colobosaura mentalis* which was later transferred to the genus *Acratosaura* by Rodrigues et al. (2009a). Burt and Burt (1933) recognized *C. kraepelini* as a valid species, a view followed by Peters and Donoso-Barros (1970) and Talbot (1979). Vanzolini and Ramos (1977) stated that the description of *C. kraepelini* is brief and not very informative so they suggested that the type specimen must be carefully analyzed to reach more solid taxonomic decisions. However, the type specimen of *C. kraepelini* (originally deposited in the Hamburg Zoological Museum) is considered to be lost (Rodrigues et al. 2007).

In this work, and in the framework of a DNA barcoding project of the Paraguayan herpetofauna, genetic and morphology data of recently collected specimens of *Colobosaura* tentatively assigned to *C. kraepelini* were analyzed, providing a redescription of its external morphology and information on its taxonomic status.

## Materials and methods

Tissue samples for genetic analyses were extracted and stored as recommended by Gamble (2014). The protocol for DNA extraction follows Ivanova et al. (2006). Samples were washed in 50 μl of diluted PBS buffer (1:9 of buffer and water respectively) for 14 h. A solution of vertebrate lysis buffer and proteinase K (60:6 μl respectively), kept at 56°C for 14 h was used for digestion. After extraction, DNA samples were eluted in 50 μL TE buffer. Amplification of mitochondrial 16S rRNA gene fragments was performed using the eurofins MWG Operon primers L2510 (forward: 5’–CGCCTGTTTATCAAAAACAT–3’) and H3056 (reverse: 5’–CCGGTCTGAACTCAGATCACGT–3’) in an Eppendorf Mastercycler pro. The PCR conditions were: denaturation 2 min (94°C) – denaturation 35 sec (94°C)×40 – hybridization 35 sec (48.5°C) – elongation 60 sec (72°C) – final elongation 10 min (72°C). The examination of DNA chromatograms and development of consensus sequences were performed with SeqTrace 0.9.0 (Stucky 2012).

The mtDNA 16S sample was compared with sequences available in GenBank for species of the most closely related clade (Iphisini: Gymnophthalminae, according to Colli et al. 2015), and a sample of *Cercosaura ocellata* (Cercosaurinae) as an outgroup. GenBank accession numbers and localities of genetic samples are provided in Appendix. It is important to note that currently the tribe Iphisini is composed of four monotypic genera (*Alexandresaurus*, *Colobosaura*, *Iphisa*, and *Stenolepis*) and two genera with two species (*Acratosaura* and *Rondonops*) (Colli et al. 2015), but we only had access to five of the eight species, missing *Acratosaura spinosa*, *Rondonops biscutatus*, and *R. xanthomystax*.

Sequences were aligned with Clustal W (Larkin et al. 2007) followed by a visual inspection and edition if necessary. Final sequence length was 512 bp. The best substitution model was chosen according to the corrected Akaike Information Criterion (AICc) (Burnham and Anderson 2002). We estimated the uncorrected genetic pairwise distances for our dataset, and performed a Maximum Likelihood (ML) analysis for a phylogenetic inference with 10,000 replicates. All these steps were executed in MEGA 6 (Tamura et al. 2013). We used FigTree v1.3.1 for tree editing (http://tree.bio.ed.ac.uk/software/figtree/).

Additionally, the external morphology of specimens of *Colobosaura* was examined (Appendix 2). We scored the following morphometric characters: snout–vent length (SVL) from the tip of the snout to the anterior edge of the cloaca; head length (HL) from the tip of the snout to the anterior edge of the ear opening; head width (HW) measured at the widest section of the head; eye diameter (ED); and ear opening (EO), both taken at the widest section. These measures (except SVL taken with a ruler) and other standard measurements were taken with digital calipers. Paired structures are presented in left/right orientation. In the color descriptions, the capitalized colors and the color codes (in parentheses) are those of Köhler (2012).

A distribution map was generated for the species of the tribe Iphisini to compare ecoregional affinities of the two species of *Colobosaura* and its closest relatives. Ecoregional information is based on Olson et al. (2001), downloaded from the web site of The Nature Conservancy (http://maps.tnc.org/gis_data.html). All coordinates are in decimal degrees and WGS 84 datum, and all the elevations are in meters above sea level. Geographic imagery produced using ArcMap 10.3. Minimum convex polygons were produced upon about 200 bibliographic records based on Brito et al. (2012) for *Acratosaura mentalis*; Rodrigues et al. (2009a) and Freitas et al. (2012) for *A. spinosa*; Freire et al. (2013) and Freitas (2014) for *Alexandresaurus camacan*; Nogueira (2001), Rodrigues et al. (2007), Cuoto-Ferreira et al. (2011), Cardozo Ribeiro et al. (2012), Freire et al. (2012), Cavalcanti et al. (2014), López Santos et al. (2014), da Silva et al. (2015), Cacciali et al. (2016), and De Alcantara et al. (2016), for *Colobosaura modesta*; Avila-Pires (1995) and Castoe et al. (2004) for *Iphisa elegans*; Colli et al. (2015) for *Rondonops biscutatus* and *R. xanthomystax*; and Rodrigues et al. (2007) for *Stenolepis ridleyi*.

Acronyms of institutions used in the text are **SMF** (Senckenberg Forschungsinstitut und Naturmuseum Frankfurt, Frankfurt am Main, Germany), **LG** (Laboratorio de Citogenetica de Vertebrados, Universidade de São Paulo, Brazil), and **MNHNP** (Museo Nacional de Historia Natural del Paraguay, San Lorenzo, Paraguay).

## Results

**Figure 1.**
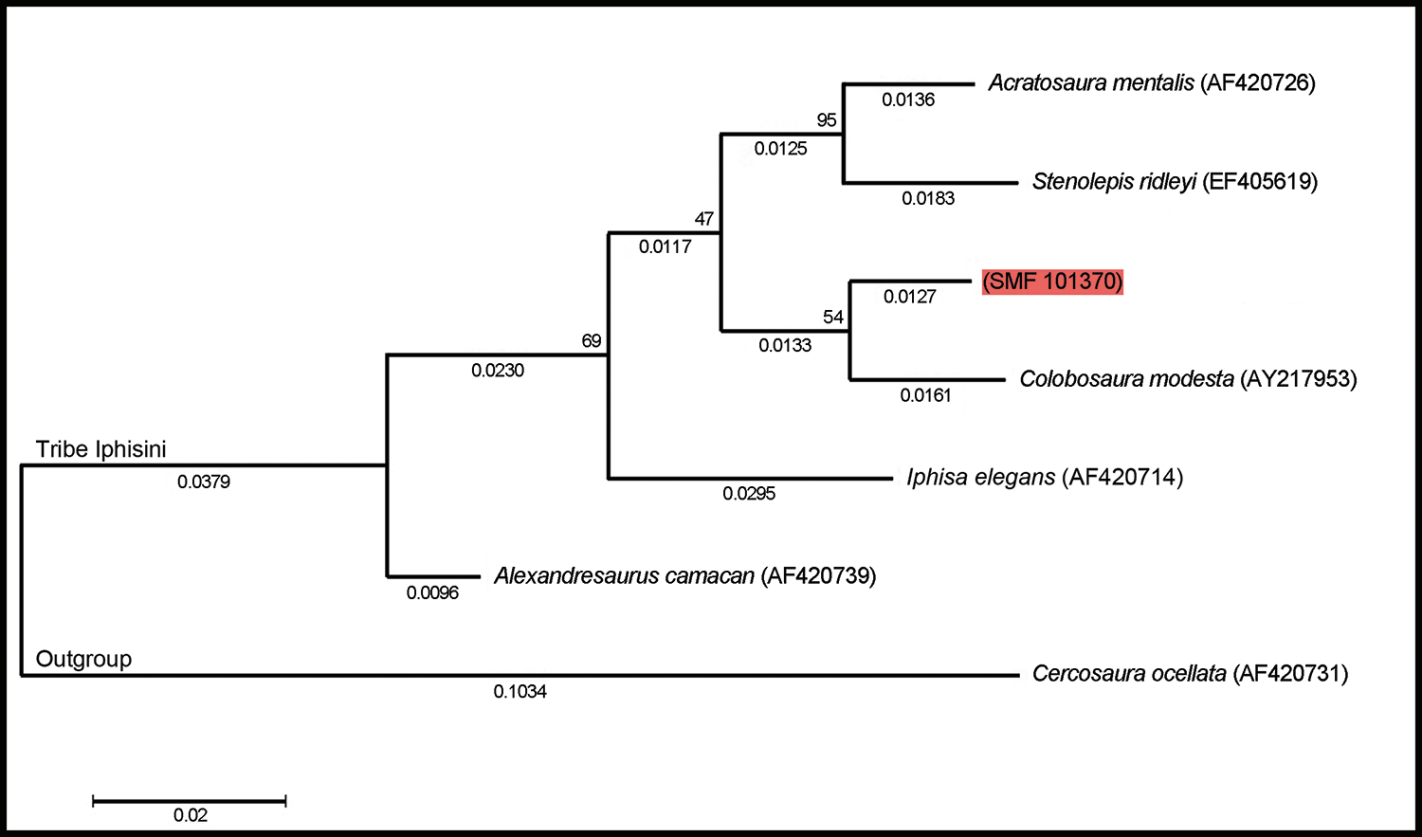
Maximum Likelihood tree obtained from 16S mtDNA for the tribe Iphisini (Gymnophthalmidae). Numbers on the nodes represent the bootstrap values and numbers below branches (and scale bar at the bottom left corner) denote branch length (substitutions/site). Specimen highlighted in red indicates our sample. See Appendix 1 for details of specimens used in the analysis.

The best substitution model was GTR+G, and the phylogeny recovered is shown in Figure 1. Our genetic sample of *Colobosaura* (SMF 101370) is sister to, but deeply divergent from *C. modesta*. A similar arrangement is observed between *Acratosaura mentalis* and *Stenolepis ridleyi* which constitute the sister clade of *Colobosaura*. *Iphisa elegans* is recovered as a sister clade of the above mentioned groups, and *Alexandresaurus camacan* as the most basal representative of the tribe.

The pairwise distance shows a divergence of ~7.7% between *C. modesta* and SMF 101370, which is even higher than the divergence between SMF 101370 and *I. elegans* (~7.1%), SMF 101370 and *S. ridleyi* (~5.5%), *C. modesta* and *S. ridleyi* (~4.7%), or *A. mentalis* and *S. ridleyi* (~3.1%) (Table 1).

**Table 1.** Pairwise genetic distances (lower-left diagonal), and SD (upper-right diagonal) among species of Iphisini: Gymnophthalminae.

	*A. mentalis*	*A. camacan*	(SMF 101370)	*C. modesta*	*I. elegans*	*S. ridleyi*
*Acratosaura mentalis*		0.016	0.016	0.013	0.015	0.001
*Alexandresaurus camacan*	0.122		0.015	0.015	0.013	0.011
*Colobosaura* (SMF 101370)	0.122	0.101		0.013	0.012	0.011
*Colobosaura modesta*	0.079	0.103	0.077		0.013	0.010
*Iphisa elegans*	0.103	0.089	0.071	0.087		0.011
*Stenolepis ridleyi*	0.031	0.055	0.055	0.047	0.060	

**Figure 2.**
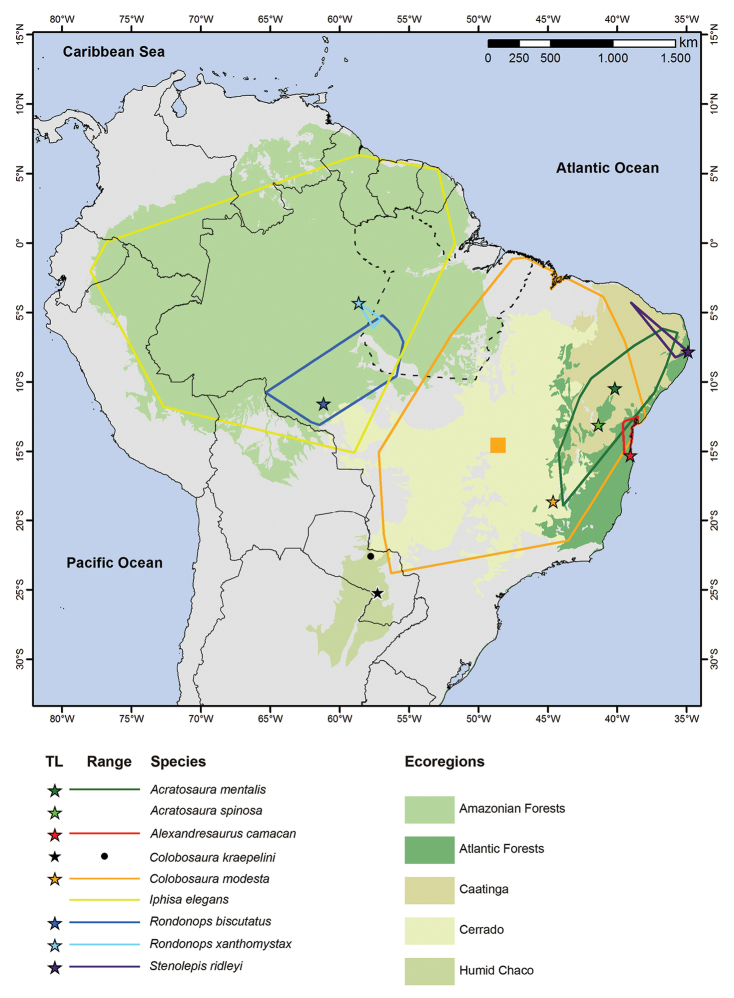
Central and northern region of South America showing the distribution (ranges in color) of the members of the tribe Iphisini. LT indicate type localities. Note that type locality for *I. elegans* is not shown since is referred as the whole Brazilian State of Pará. Range for *A. spinosa* is not shown because records come from vicinities of type locality. *Colobosaura kraepelini* is known only from two areas: the locality mentioned in the original description (black dot) and the neotype locality (black star); the second specimen of *C. kraepelini* reported here is from near the neotype locality. Orange square represents locality of the genetic sample of *C. modesta* (Niquelândia, GO, Brazil). Data for ecoregions according to Olson et al. (2001).

From the distribution it is possible to identify two groups within the tribe Iphisini: one strongly related to Amazonian ecoregions (*Iphisa* and *Rondonops*), and another linked to the Dry Diagonal (*Acratosaura*, *Alexandresaurus*, *Colobosaura*, and *Stenolepis*). Two monotypic genera (*Alexandresaurus* and *Stenolepis*) and *Acratosaura spinosa* are mainly associated to Caatinga environments, whereas *Acratosaura mentalis* have some records in Cerrado. *Colobosaura modesta* together with *Iphisa elegans* has the widest distribution, and it is strongly linked to Caatinga and Cerrado. The collecting site of SMF 101370 is in the Humid Chaco (Fig. 2).

The genetic data presented above demonstrate that our sample SMF 101370 is not conspecific with *C. modesta*. The only other available nominal species that SMF 101370 could be assigned to is *Colobosaura kraepelini* Werner, 1910. Unfortunately, the holotype and only known specimen of this taxon is considered to be lost (see above) and its original description is brief. Therefore, there is no morphological basis to support our claim that SMF 101370 is conspecific with *C. kraepelini* which leaves us with two options: The more conservative option is to assign SMF 101370 to *C. kraepelini* whereas the alternative would be to describe a new species based on our sample. Since we know of no diagnostic character that would differentiate between SMF 101370 and *C. kraepelini*, we think that the better option is to assign SMF 101370 to *C. kraepelini*. Thus, we herewith designate SMF 101370, a subadult male from 2.5 km E of Altos (25.2588°S, 57.2850°W, ca 280 masl), Cordillera Department, Paraguay, collected on 27 February 2012 by Gunther Köhler, as the neotype of *C. kraepelini*. Thereby we clarify and stabilize this taxonomic situation and link the name *kraepelini* to a voucher specimen and a genetic sample which will help to avoid taxonomic uncertainties in the future. We provide a species account and description of the neotype as well as data on individual variation below.

### Colobosaura kraepelini

Taxon classificationAnimaliaSquamataGymnophthalmidae

Werner, 1910

**Figure 3.**
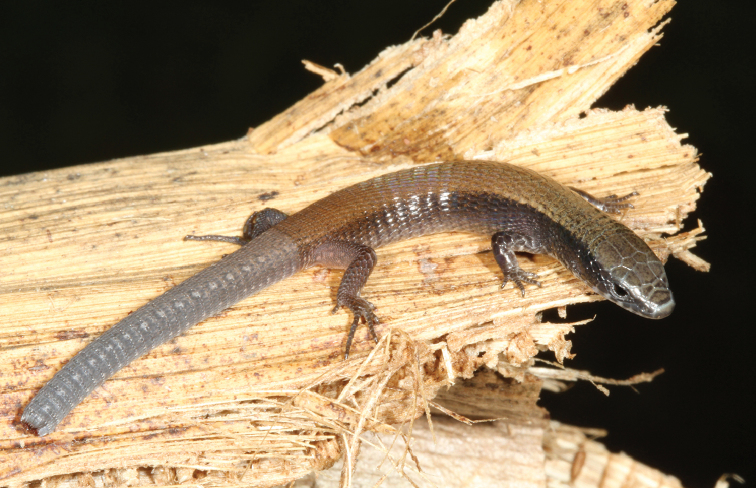
Neotype of *Colobosaura kraepelini* (SMF 101370) from the vicinity of Altos, Cordillera Department, Paraguay.

Colobosaura kraepelini Werner, 1910: 32 (neotype, SMF 101370 [by present designation] (Fig. 3); type locality: 2.5 km E of Altos (25.2588°S, 57.2850°W, ca 280 masl), Cordillera Department, Paraguay by neotype selection). Original type locality: Puerto Max, San Pedro Department, Paraguay.

#### Diagnosis.

**Figure 4.**
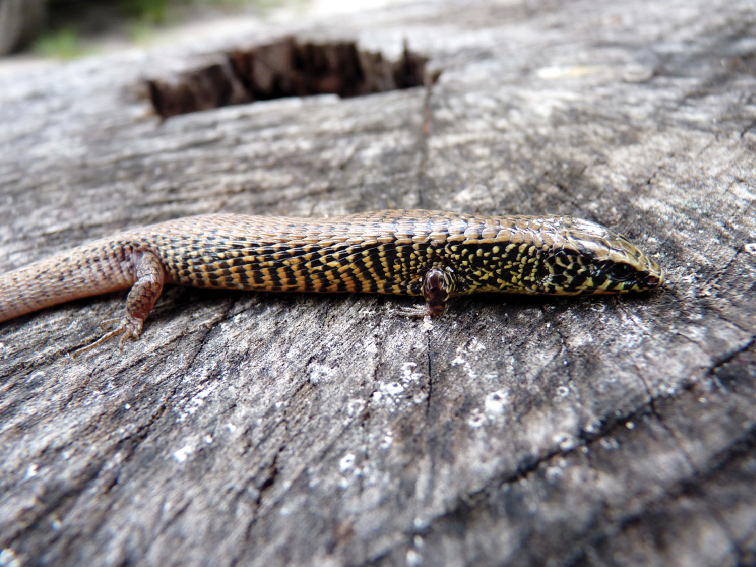
Specimen of *Colobosaura modesta* showing lateral coloration patter. Image given by Paul Smith (Fauna Paraguay). Additional photographs available at http://www.faunaparaguay.com/colobosauramodesta.html

**Figure 5.**
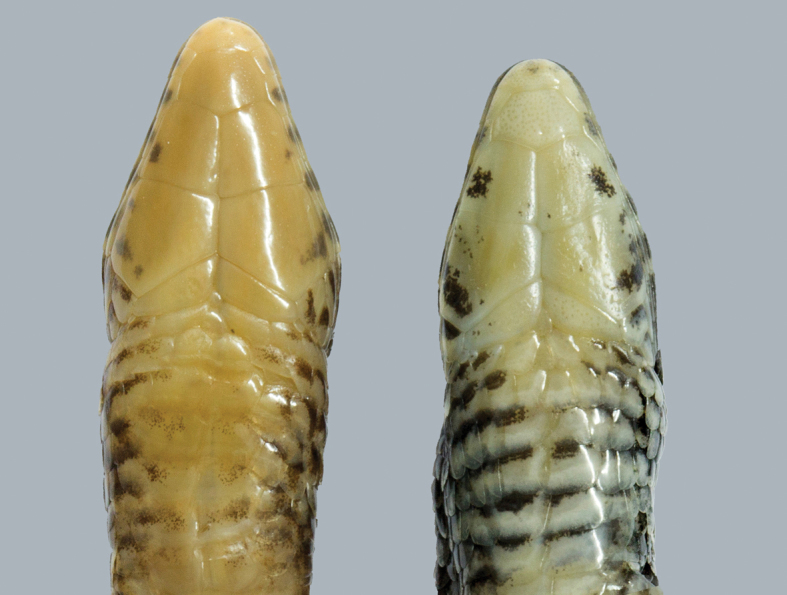
Ventral view of the head showing the different coloration pattern between *Colobosaura modesta* (MNHNP 8521, left) and *C. kraepelini* (MNHNP 11726, right).

*Colobosaura kraepelini* differs from the other species of the family Gymnophthalmidae except for *C. modesta*, by a combination of the following characters: limbs short but well developed; Finger I vestigial, not clawed; dorsal and lateral body scales keeled; four longitudinal series of ventral scales; prefrontal present; occipital present; two pairs of chin shields. *Colobosaura kraepelini* differs from *C. modesta* by having two mid-central rows of immaculate scales (vs. four immaculate ventral rows in *C. modesta*); flanks completely dark (Fig. 3) (vs. clear mottling in that area in *C. modesta*, Fig. 4); and gular shields profusely suffused with dark reaching the midline (vs. dark mottling restricted to the external edge of the shields, Fig. 5).

*Description of the neotype.* Subadult male. Body elongated; neck not well differentiated; SVL 29 mm; tail (clipped) stump 14 mm; HL 6.55 mm; HW 4.52 mm; ED 1.42 mm; EO (oblique) 0.66 mm. Head with juxtaposed scales, except posterior edge of interparietal and parietals imbricate with occipital and first row of nuchal scales.

Rostral broad, wider (1.81 mm) than high (0.72 mm), contacting frontonasal, nasals, and first supralabials; frontonasal heptagonal, wider (1.81 mm) than long (1.30 mm), contacting rostral, nasals, loreals, and prefrontals; prefrontals wider (1.07 mm) than long (0.70 mm) with a 0.29 mm contact line between them, and contacting frontonasal, loreals, first and second supraocular, and frontal; frontal hexagonal, longer (1.67 mm) than wide (1.11), contacting prefrontals, second supraocular, and frontoparietals; frontoparietals regular pentagonal, with a 0.67 mm mid contact line between them, and contacting frontal, second (slightly) and third (broad contact) supraoculars, parietals, and interparietal; interparietal longer (2.15 mm) than wide (1.18 mm), contacting frontoparietals, parietals, first row of nuchals, and occipital; parietals broad, wider than interparietal, contacting the interparietal, frontoparietals, third supraocular, three rows of temporals, and the first row of nuchals; occipital pentagonal and small (0.57×0.83 mm) located between the interparietal and the first and second row of nuchals; nasal elongated (0.95×0.72 mm), with nares located in the mid-lower region, contacting the rostral, frontonasal, loreals, and first supralabial; loreal curved, higher (0.67 mm) than wide (0.41 mm), in contact with nasal, frontonasal, first supraocular, first superciliary, preocular (narrowly), frenocular, and first (slightly) and second superciliaries; of which the middle one is the shortest; three supraoculars, the first smaller than the other two; three elongated superciliars, being the middle scale shorter than the first and third; eleven upper palpebrals and ten lower palpebrals surrounding the orbit; semitransparent eyelid; four elongated suboculars, second and third longer than first and fourth; seven supralabials, first contacting rostral, nasal, and loreal narrowly; second contacting loreal, frenocular, and the first subocular; third and fourth supralabials in contact with suboculars; fifth supralabial (largest) contacting third and fourth subocular, lower postocular, and lower first temporal, sixth contacting the lowermost scale of the second temporal row, and other scales in the temporal region, and seventh supralabial reaching the border of the ear opening; two postoculars, the upper (in contact with the two last upper palpebrals, third superciliary, third supraocular, and upper temporal) slightly larger than the lower (in contact with the last upper palpebral, fourth subocular, fifth supralabial, and the first row of temporals); two first temporals, the upper twice the size of the lower; three second temporals, the upper twice longer than the two lower.

Mental broad, wider (mm) than long (mm); postmental pentagonal, wider (mm) than long (mm), in contact with mental, first and second infralabials, and first pair of chin shields; two pairs of chin shields, the second larger than the first pair, and followed by elongated and oblique scales that separate the second pair of chin shields from the scales of the gular region; seven infralabials, the first the widest, and the fifth the longest.

Nuchal region with seven rows of paired imbricate scales; lateral sides of the neck with three to four irregular series of juxtaposed scales, and two imbricate located in the lowermost portion; seven paired rows of gular scales, first two rows irregular, and homogeneously arranged in pairs from the third to the seventh row.

Dorsal scales imbricate, 21 transversal rows between axilla and groin, wider at neck level, and narrower and homogeneously arranged in longitudinal rows on trunk; lateral scales similar to dorsals in the upper flanks, becoming wider towards the ventral region; sternal scale triangular, flanked by large rectangular scales in the clavicular region; four longitudinal rows of ventral scales; 26 scales around midbody; scales at insertion of limbs granular, except in the ventral region; all of tail with imbricate, elongated, hexagonal, and keeled scales.

Forelimbs covered with large, imbricate and smooth scales on the dorsal and lateral surfaces, being smaller on the ventral region of the limb; carpal region covered with large imbricate scales; palmar surface covered with granular juxtaposed scales; scales on fingers from I to V: 1/1-4/5-6/6-7/7-4/4; infradigital single lamellae under fingers from I to V: 2/2-8/8-10/10-11/12-6/5; fingers clawed except vestigial finger I.

Hind limbs medium-sized, imbricate, moderately keeled scales on the dorsal surface; anterior and posterior parts of the hind limbs with large, imbricate, and smooth scales; posterior part of hind limbs covered with granular juxtaposed scales on the thigh, and smooth medium-sized imbricate scales on the shank; tarsal region covered with large imbricate scales; plantar surface covered with granular juxtaposed scales; scales on toes from I to V: 3/3-4/4-8/8-10/10-6/(toe clipped as tissue sample); infradigital single lamellae under toes from I to V: 4/4-8/7-14/12-15/17-9/(toe clipped); toes clawed.

#### Coloration in life of the neotype.

Dorsal surface of head Olive Clay Color (85) with Vandyke Brown (282) mottling on frontal and second supraocular and posteriorly, and a diffuse Vandyke Brown (282) line edging anterior margin of frontal and second supraocular and anterior scales; lateral parts of the head homogeneous Vandyke Brown (282); supralabials with Medium Neutral Gray (298) bars in the center interleaved with Cyan White (155) in the sutures; background color of mandibular region Cyan White (155) with Medium Neutral Gray (298) blotches on infralabials (one per scale) and second pair of chin shields; iris Burnt Umber (48); dorsal scales Mikado Brown (42), anteriorly (before forelimbs level) with Vandyke Brown (282) suffusions more concentrated near the laterals, and posteriorly (after forelimbs level) with faint irregular suffusions of Warm Sepia (40), more regularly present on the scales margins; lateral sides of the neck and body Vandyke Brown (282) with irregular Mikado Brown (42) speckles and blotches after forelimbs level, grading into a reticulated Vandyke Brown (282) and Mikado Brown (42) pattern near the groin; background ventral color Cyan White (155) with intrusions of Vandyke Brown (282) on the throat, and a faint mottling of Vandyke Brown (282) on the lateral rows of ventral scales; forelimbs mostly Vandyke Brown (282), Cyan White (155) restricted to the anteroventral regions; hind limbs Mikado Brown (42) with suffusion of Vandyke Brown (282) on the scales margins, and Cyan White (155) on the ventral region of the limb; tail background color Plumbeous (295) with Brownish Olive (292) suffusions on the anterior third of the organ, and Pale Greenish White (97) paravertebral spots located every two scales; iridescent hue all along the body.

#### Coloration in preservative of the neotype.

(After five years in 70% ethanol): The general pattern remains the same, and the background Mikado Brown (42) color also remains; the darker parts of the body (lateral sides of neck and body) turned to Sepia (279); tail turned to Hair Color (277) on the dorsum, with the paravertebral spots faintly visible; ventral side of the head Smoky White (261); ventral side of the body Pale Buff (1).

#### Variations.

MNHNP 11726 agrees well in most aspects of the scalation to those observed in the neotype, with the following differences: two superciliaries; 21 transversal rows between axilla and groin; 27 scales around midbody; 11 infradigital lamellae under IV finger; 16 infradigital lamellae under IV toe. Background color of MNHNP 11726 slightly clearer (Sayal Brown 41) than SMF 101370, and the dark (Fuscous 283) lateral suffusions are less dense. Ventrally Pale Buff (1). The coloration pattern is the same in both specimens with some differences: MNHNP 11726 has dark blotches also on the first pair of chinshields; posterior margin of dorsal scales strongly marked; caudal spots absent.

#### Distribution and habitat.

The species is distributed in the Humid Chaco. The environment is basically a savanna composed of palms (*Copernicia alba*), native bunch grasses, and scattered islands of semideciduous temperate forest. The area is adapted to periodical floods from the Paraguay River. The locality of Puerto Max (former type locality of *C. kraepelini*) consists of a small village and cattle farm with intense anthropic pressure. The new specimens (SMF 101370 and MNHNP 11726) came from the vicinities of the capital city, about 280 km (airline) southwards from the original type locality, also in Humid Chaco.

## Discussion

The tribe Iphisini was described recently by Rodrigues et al. (2009b) which was before merged within the tribe Heterodactylini. Nevertheless, Rodrigues et al. (2007) already discovered that the genera *Acratosaura*, *Alexandresaurus*, *Colobosaura*, *Iphisa*, and *Stenolepis* exhibit a strong sexual dimorphism, absent in other Heterodactylini. Our ML phylogenetic hypothesis of the tribe Iphisini based on the mtDNA 16S gene recovered *Acratosaura mentalis* and *Stenolepis ridleyi* as sister taxa which was also inferred by Rodrigues et al. (2007) and Colli et al. (2015). The position of *Iphisa* differs from the phylogeny presented by Colli et al. (2015), being the sister clade of *Acratosaura*+*Colobosaura*+*Stenolepis* in our analysis. It is important to note that sequences of *Rondonops biscutatus* used by Colli et al. (2015) were not available at GenBank. The placement of *Iphisa* as a basal clade in relation to *Acratosaura* and *Colobosaura* was also shown by Pellegrino et al. (2001) and Castoe et al. (2004). And *Alexandresaurus camacan* is shown as the most basal taxon in the group (Fig. 1) as also exposed by Pellegrino et al. (2001), Castoe et al. (2004) (referred in these two publications as *Colobosaura* spn), Rodrigues et al. (2007), and Colli et al. (2015).

From the genetic point of view there is no doubt that the neotype of *Colobosaura kraepelini* is different from *C. modesta*. The high genetic distance between these two species compared with the even lower genetic distance between some related genera (Table 1) could indicate that a new taxonomic arrangement should be proposed. Nevertheless, based on the little morphological differentiation in *Colobosaura* we keep a conservative approach. In our phylogeny, the divergence between *Colobosaura modesta* and *C. kraepelini* is as deep as the divergence between the genera *Acratosaura* and *Stenolepis*.

The only previously known reference to a specimen of *Colobosaura kraepelini* was in the original description based on an individual from Puerto Max, and the species was never found again. Given the brevity of the original description the species was considered as synonym of *C. modesta* (Vanzolini and Ramos 1977, Rodrigues et al. 2007). Vanzolini and Ramos (1977) additionally stated that maybe the specimen used for the description of *C. kraepelini* was not even a *Colobosaura* because in the description the author referred to some oblique folds on the tongue of the specimen, which is a character that does not occur in the group. Our specimen differs morphologically from *C. modesta* in some aspects of coloration, and it was found in the Humid Chaco (as is the original type locality of *C. kraepelini*) whereas *C. modesta* is restricted to Caatinga and Cerrado in areas adjacent to Atlantic Forest (Fig. 2). All three known localities for *C. kraepelini* are located in the drainage system of the Paraguay River sharing some topographical traits.

Biogeographically, Rodrigues et al. (2007) hypothesized that *Stenolepis* should have originally a wider distribution followed by a major constriction, resulting in its current restricted range associated with the Atlantic Forest. The basal location of *Alexandresaurus* in the tribe’s phylogeny could suggest that it probably also had a wider distribution, although it is currently restricted to a small patch of Atlantic Forest on the coast of Bahia. In the remaining taxa it is possible to distinguish a major phylogenetic split of eastern (only *Iphisa* in our phylogeny) and western (*Acratosaura*, *Colobosaura*, and *Stenolepis*) clades, which was also noted by Colli et al. (2015). Whereas the western clade is strictly related to Amazonian forests, the eastern clade is present mainly in the Dry Diagonal, although *S. ridleyi* is also present in Atlantic Forest and Caatinga (Fig. 2). According to this biogeographical perspective and based on the distribution of the whole tribe, *C. kraepelini* could be the most derived member of the clade.

Rodrigues et al. (2007) highlighted the importance of analyzing the wide distribution ranges of *Colobosaura* and *Iphisa* and, in fact, more recently Nunes et al. (2012) revealed that *Iphisa* is actually composed of five different species, and Colli et al. (2015) suggest that a detailed analysis of *Colobosaura* could indicate a similar pattern. Here we provide evidence that at least the genus *Colobosaura* is composed of two species. The morphological traits proposed by Peters and Donoso-Barros (1970) to differentiate between *C. modesta* and *C. kraepelini* (shape of the interparietal) are useless. Instead, we show that coloration can differentiate between these two taxa. Following, we present a key for the identification of species in the tribe Iphisini.

### Key to species of Iphisini

**Table:** 

1	Two longitudinal rows of ventral scales	**2**
–	Four or six longitudinal rows of ventral scales	**4**
2	One pair of enlarged chin shields	***Iphisa elegans***
–	Two pairs of enlarged chin shields	(***Rondonops***) **3**
3	Lateral neck scales smooth; 16–20 infradigital lamellae under toe IV	***R. biscutatus***
–	Lateral neck scales keeled; 20–26 infradigital lamellae under toe IV	***R. xanthomystax***
4	Prefrontals absent	***Stenolepis ridleyi***
–	Prefrontals present	**5**
5	Occipitals absent	***Alexandresaurus camacan***
–	Occipitals present	**6**
6	Three pairs of chin shields	(***Acratosaura***) **7**
–	Two pairs of chin shields	(***Colobosaura***) **8**
7	Lateral neck scales smooth and juxtaposed; dorsal scales slightly keeled (keel covers half of the scale) at midbody	***A. mentalis***
–	Lateral neck scales keeled and imbricate; dorsal scales strongly keeled at midbody	***A. spinosa***
8	Ventrals immaculate; dark mottling on the external edge of gular shields	***C. modesta***
–	Two central rows of ventral scales immaculate, and dark mottling on the two external rows; gular shields profusely mottled with dark	***C. kraepelini***

## Supplementary Material

XML Treatment for Colobosaura kraepelini

## Appendix 1

### Genetic samples

**Table:**

Species	Voucher	GBAN	Locality
*Acratosaura mentalis*	MRT 906448	AF420726	Morro do Chapéu, BA, Br
*Alexandresaurus camacan*	MD 1106	AF420739	Una, BA, Br
*Colobosaura kraepelini*	SMF 101370	KY782646	Altos, Cordillera, Pa
*Colobosaura modesta*	LG 1145	AY217953	Niquelândia, GO, Br
*Iphisa elegans*	MRT 977426	AF420714	Aripuanã, MT, Br
*Stenolepis ridleyi*	-?-	EF405619	-?-
*Cercosaura ocellata* OG	MRT 977406	AF420731	Aripuanã, MT, Br

For each species the voucher specimen, GenBank accession number (GBAN), and the locality (Br = Brazil, Pa = Paraguay) are presented for samples used in the genetic analysis. Outgroup marked with OG. See Materials and methods section for indication of institutional acronyms. MRT and >MD indicate Miguel Trefaut Rodrigues (Museu de Zoologia, Universidade de São Paulo, Brazil) and Marianna Dixo (Instituto de Biociências, Universidade de São Paulo, Brazil) voucher specimens, respectively. Data for the sample of *S. ridleyi* are missing in the original publication (Rodrigues et al. 2007).

## Appendix 2

### Examined specimens

*Colobosaura kraepelini*

**PARAGUAY**: Cordillera: San Bernardino, 50 metros del Lago Ypacarai (MNHNP 11726).

*Colobosaura modesta*

**PARAGUAY**: Amambay: Parque Nacional Cerro Corá (MNHNP 8454–56, 8521). San Pedro: Reserva Natural Laguna Blanca (MNHNP 11684, 11596, 11652).
